# Effectiveness of Propolis in the Treatment of Periodontal Disease: Updated Systematic Review with Meta-Analysis

**DOI:** 10.3390/antiox10020269

**Published:** 2021-02-10

**Authors:** Nansi López-Valverde, Beatriz Pardal-Peláez, Antonio López-Valverde, Javier Flores-Fraile, Silvia Herrero-Hernández, Bruno Macedo-de-Sousa, Julio Herrero-Payo, Juan Manuel Ramírez

**Affiliations:** 1 Department of Surgery, Instituto de Investigación Biomédica de Salamanca (IBSAL), University of Salamanca, 37007 Salamanca, Spain; nlovalher@usal.es (N.L.-V.); bpardal@usal.es (B.P.-P.); j.flores@usal.es (J.F.-F.); silvia_herrero@usal.es (S.H.-H.); jhpayo@usal.es (J.H.-P.); 2 Polo I-Edifício Central Rua Larga, Institute for Occlusion and Orofacial Pain Faculty of Medicine, University of Coimbra, 3004-504 Coimbra, Portugal; brunomsousa@usal.es; 3 Department of Morphological Sciences, University of Cordoba, Avenida Menéndez Pidal s/n, 14071 Cordoba, Spain; jmramirez@uco.es

**Keywords:** propolis, periodontal disease/periodontitis, gingivitis, oral health, oxidative stress

## Abstract

In recent times, the use of natural products has gained momentum, either as a treatment or as adjuvants for other drugs in the treatment of different conditions. Propolis is a natural substance produced by bees which has proven useful for treating periodontal disease. This systematic review and meta-analysis gather evidence of the effectiveness of propolis in this kind of condition. The MEDLINE, CENTRAL, PubMed, EMBASE and Web of Science databases were searched for scientific articles to identify the findings published up to October 2020. The MeSH phrases used in the search were: “periodontal diseases AND propolis treatment”; “gingivitis AND propolis treatment”; “periodontitis AND propolis treatment”; “propolis treatment AND oral health”; “propolis AND oxidative stress AND periodontitis”. The Boolean operator “AND” was used to combine the searches. Randomized trials where propolis was used in the treatment of different periodontal conditions were included. Non-randomized clinical studies were systematically reviewed and 224 studies were detected, eight of which met the criteria for inclusion in the meta-analysis. Only three of these were selected for quantitative synthesis. In conclusion, propolis is safe to use and can improve the results of periodontal disease treatment, reducing probing pocket depth compared with treatment with a placebo (difference in means, fixed effects −0.67 [95% CI: −0.84, −0.50]).

## 1. Introduction

Propolis is a non-toxic resinous substance produced by bees that has antimicrobial, antifungal, anti-inflammatory, antioxidant and antitumor properties, among others [1,2], which have attracted the attention of researchers, both in the medical and in the dental field.

It is a mixture of plant extracts mixed with the bees’ own saliva, which varies according to its place of origin, with Brazil leading the research on this product. Its composition is based on vegetable resins (50%), waxes (30%), essential and aromatic oils (10%) and pollen and other organic substances (10%). The chemical composition of propolis depends on the geographic location, the botanical origin and the species of bee [3,4]. Nevertheless, its composition is quite complex, its main components being flavonoids and phenolic esters such as caffeic acid phenethyl ester [5,6]. The flavonoids contained in propolis have been found to have antimicrobial, anti-inflammatory and immunomodulating properties, which are extremely useful to treat aphthous ulcers, candidiasis, gingivitis and periodontitis [7,8].

Periodontal disease is likely to be the most common chronic infection in adults [9]. There is evidence that suggests that nearly all forms of periodontal disease are specific chronic bacterial infections that stem from the overgrowth in dental plaque of a limited number of species, mainly anaerobic, such as *Porphyromonas gingivalis*, *Bacteroides forsythus* and *Treponema denticola* [10], considering the induction of inflammatory signaling pathways by pathogenic bacteria to be crucial for the development of inflammatory processes in the periodontium [11].

Periodontal diseases have traditionally been associated with the development of certain systemic conditions, systemic inflammation being one of the main reasons for this association [12].

The presence of inflammatory infiltrate and an increase in the oxidative response of peripheral polymorphonuclear cells, are constant characteristics of periodontal disease, there being greater damage as the disease advances because of the increase in the number of reactive oxygen substances (ROS). The increase in the amount of free radicals, causes oxidative damage to gingival tissue, periodontal ligament and alveolar bone. The deterioration caused by free radicals is regulated by an antioxidant defense system, in such a way that when an imbalance between the production of free radicals and antioxidant levels takes place, it triggers a condition known as oxidative stress (OS), which is defined as an imbalance between the production of free radicals and the body’s capacity to eliminate these reactive species [13,14,15].

Free radicals are extremely unstable organic and inorganic molecules that contain an unpaired electron. They have the capacity to take electrons from other atoms and molecules, causing a release of proinflammatory cytokines such as interleukin-2, 6 and 8 (IL-2, IL-6 and IL-8), interferon-β and tumor necrosis factor α (TNF-α), which play an extremely relevant role in the pathogenesis of periodontal disease [16,17].

It is believed that disturbances in the local and/or general indicators of oxidative stress are one of the mechanisms in the etiopathogenesis of periodontitis, and, in recent years, different basic clinical and experimental studies have provided evidence of a strong association between oxidative stress and periodontal disease [18].

A better understanding of this association could provide deeper insight into the pathology of this disease, shed further light on the relationship between periodontal disease and systemic inflammation, and increase knowledge of therapeutic approaches.

As systematic reviews are an essential tool to synthesize the available scientific information and identify areas of uncertainty where research is crucial, the purpose of this study was to conduct a systematic review of the literature on the effectiveness of propolis in the treatment of periodontal disease.

Meta-analysis (where possible) provides very useful information that facilitates understanding of the effect of a treatment in specific patient groups. Likewise, it enhances accuracy in the estimation of a certain effect, identifying moderate but clinically important degrees that could go unnoticed in primary studies. This meta-analysis was carried out using randomized clinical trials (RCTs) on the use of propolis in the treatment of periodontal disease.

## 2. Materials and Methods

The study selection process was carried out according to the Preferred Reporting Items for Systematic Review and Meta-Analyses (PRISMA) guidelines for systematic reviews and meta-analysis [19] (Table S1 Checklist).

### 2.1. Protocol

The search strategy was conducted using the population, intervention, comparison and outcome (PICO) framework, based on the following question:

“Is propolis effective in the treatment of periodontal disease?”

To answer this question, a sample of patients with periodontal disease, regardless of age, was selected. Treatment consisted of the use of propolis products, such as mouthwashes/gels or capsules, alone or complementary to other non-surgical therapies. Control patients were treated using conventional treatments, placebos or other types of mouthwash/gel. The results reviewed in the literature were plaque index, gingival indices, periodontal indices and microbiological parameters.

### 2.2. Data Sources and Search Strategy

The MEDLINE, CENTRAL, PubMed, EMBASE and Web of Science electronic databases were searched for findings published until October 2020. The MeSH phrases (Meaning of MeSH: Medical Subject Headings) used in the search were: “periodontal diseases AND propolis treatment”; “gingivitis AND propolis treatment”; “periodontitis AND propolis treatment”; “propolis treatment AND oral health”; “propolis AND oxidative stress AND periodontitis”. The Boolean operator “AND” was used to combine and narrow the searches.

### 2.3. Inclusion and Exclusion Criteria

Inclusion criteria:

(a) Articles published in English,
(b) Randomized controlled clinical trials,
(c) Non-randomized studies assessing the effectiveness of propolis in the treatment of periodontal diseases.

Exclusion criteria:

(a) In vitro studies,
(b) Animal studies,
(c) Comparative studies,
(d) Systematic reviews,
(e) Clinical cases,
(f) Non-relevant studies (e.g., effectiveness of propolis in the treatment of other conditions, narrative reviews…), duplicate studies and those that did not meet the inclusion criteria stated above.

### 2.4. Data Extraction and Analysis

Studies that did not refer to the research question were removed, and the titles and abstracts of the selected articles were collected and entered in an Excel spreadsheet. Two reviewers (NL-V and AL-V) selected the titles and abstracts independently. Disagreements regarding study inclusion were resolved through discussions between the two reviewers mentioned. Subsequently, the full texts of the selected studies were obtained for review and inclusion. The bibliographical references of each study were reviewed as possible sources for finding additional studies.

### 2.5. Quality of the Reports of the Included Randomized Trials

This was assessed using the Jadad scale [20], which defines the methodological quality of the studies based on their description of randomization, blinding and withdrawals (dropouts). The scale ranges from 0 to 5, a score ≤ 2 meaning low report quality and a score ≥ 3 meaning high report quality (Table 1).

### 2.6. Statistical Analysis

The meta-analysis was performed using RevMan 5 software (Review Manager (RevMan) [computer program], version 5.3; Copenhagen, The Nordic Cochrane Centre, The Cochrane Collaboration, 2014). Difference in means (DM) and standard deviation (SD) were used to assess continuous variables (probing depth) with a 95% confidence interval (CI). The threshold for statistical significance was *p* < 0.05. While the possibility of performing a quantitative analysis of the different parameters measured in the studies was considered, it was only possible to carry out the meta-analysis of probing depth assessment. Regarding the rest of the parameters assessed in the studies, the Oral Hygiene Index (OHI) was evaluated in 2 of the studies [24,25] and the Gingival Index (GI) in another 2 [25,28]. Although gingival bleeding was measured in most of the studies, it was tested using different indices depending on the study, which created a disparity of clinical criteria for assessment and precluded the unification of data [21,22,23,26,27,28]. As for plaque control, 4 studies tested it using the Plaque Index (PI) [23,24,25,26], although 2 of them [25,26] did not provide numerical data. Concerning clinical attachment level, although it was measured in 3 of the studies [21,22,28], that by Sparabombe and colleagues [22] did not explain the measurement criterion, which means that it was discordant with the measurement units used in the other 2 studies. Because of this, it was determined that the only parameter that was tested with unity of criterion among the studies selected was probing pocket depth (PPD), measured in mm and examined in 6 of the 8 studies selected for the meta-analysis [21,22,23,25,26,28].

## 3. Results

### 3.1. Characteristics of the Studies

**Figure 1:**
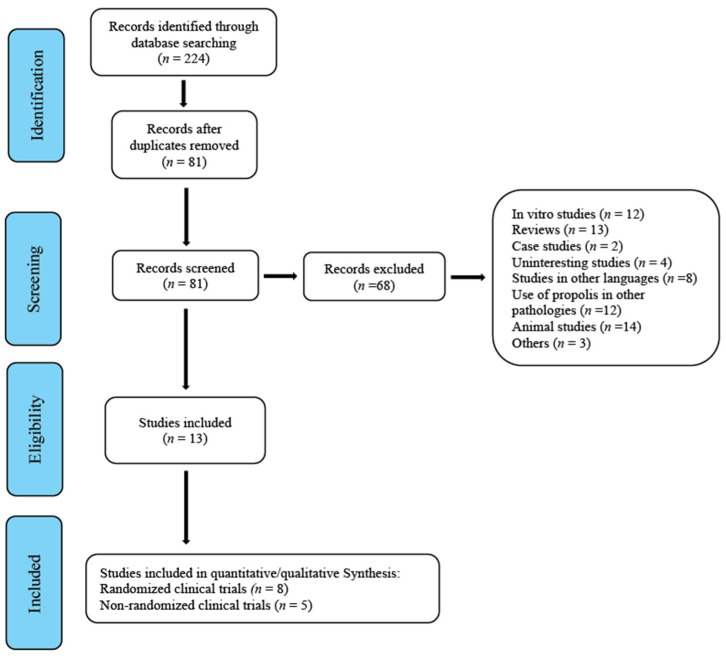
Flowchart.

Until October 2020, 224 studies were gathered and subsequently assessed by the reviewers. The first screening led to the removal of 143 duplicates. In a second screening, 65 studies that did not clearly meet the inclusion criteria and were therefore considered inadequate were removed. After this, three more were removed for different reasons: one because it was a review/commentary [29], one that tested the efficacy of propolis in reducing chemotherapy-induced oral mucositis [30] and another [31] because it was study of equivalence conducted on induced gingivitis. This left a total of 13 studies: eight randomized clinical trials to be included in the meta-analysis [21,22,23,24,25,26,27,28] and five non-randomized studies [32,33,34,35,36] (Figure 1, flowchart).

Table 2 provides a general description of the details of the randomized studies. The five clinical trials [32,33,34,35,36] that were not relevant to our meta-analysis were systematically reviewed.

### 3.2. Methodological Quality of the Included Randomized Studies

All the studies included in the meta-analysis reached a Jadad scale score that was compatible with high methodological quality (≥3 points), the study of Nakao and colleagues [21] achieving the highest score (Table 1).

### 3.3. Results of the Meta-Analysis

Meta-analysis was carried out to evaluate probing pocket depth at 3 months from the beginning of treatment with propolis or with a placebo, according to the tested group [21,22,23,25,26,28]. The study by Sharkawy and colleagues [26] was excluded from the meta-analysis because although it measured probing depth, it did not include numerical data. The studies by Giammarinaro and colleagues [23] and Shangani and colleagues [28] were also excluded on the grounds that they used chlorhexidine rather than a placebo with the control group to carry out the comparison and did not perform probing 3 months after the beginning of the trial. Thus, only three studies were eligible for quantitative synthesis [21,22,25].

Low heterogeneity among studies (I^2^ = 3%, 95% CI) led to the selection of a fixed effects model, assuming that the differences among studies were not the result of heterogeneity but of random effects.

A forest plot was used to check that the difference between both treatments in individual studies was not significant in the studies by Pérez de Andrade and colleagues [25] and Sparabombe and colleagues [22], since the 95% confidence intervals overlapped and crossed the line of no effect. In the study by Nakao and colleagues [21], the experimental group treated with propolis yielded better results than the group treated with a placebo. Moreover, this study achieved a weight of 95% in the meta-analysis.

**Figure 2:**
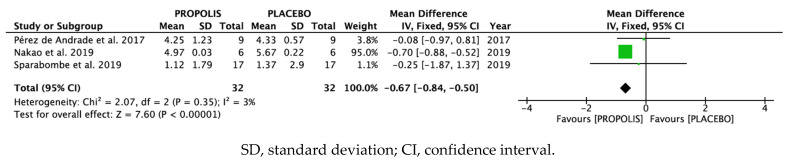
Forest plot.

According to the overall results of the meta-analysis, treatment with propolis reduced probing pocket depth as compared with treatment with the placebo (difference in means, fixed effects –0.67; 95% CI: −0.84, −0.50) (Figure 2).

### 3.4. Publication Bias and Heterogeneity

The low number of selected studies precluded the evaluation of publication bias using a funnel plot.

### 3.5. Results of Systematic Review

A systematic review was conducted to summarize the clinical trials that were not eligible for meta-analysis because they were not randomized [32,33,34,35,36]. One of the studies, which tested the use of propolis-based toothpaste on a sample of 30 individuals aged 7 to 12 once a day for a 4-month period [32], reported an exponential reduction in salivary microbial load from the beginning of the treatment. Three studies [33,34,35] evaluated dental plaque and anaerobic bacteria in patients treated with propolis solutions and placebos. All the studies reported the benefits of propolis regarding the Plaque Index (PI) and Oral Hygiene Index (OHI), with a ≥25% reduction; only the study by Gebaraa and colleagues [36], based on a sample of 20 patients diagnosed with chronic periodontitis and treated with propolis or placebo, revealed no statistically significant differences between groups in terms of Plaque Index (PI), Gingival Index (GI) and Clinical Attachment Level (CAL). Nevertheless, these authors had previously reported the in vitro antimicrobial efficacy of propolis using propolis extracts in serial concentrations [37]. Table 3 provides a summary of the characteristics and results of these trials.

### 3.6. Assessment of Non-Randomized Clinical Trials

These were evaluated using the Newcastle–Ottawa Scale (NOS). Recommended by the Cochrane Non-Randomized Studies Methods Working Group [38], this is an instrument developed to assess the quality of non-randomized studies. Each study was assigned a score of 0–9. Studies that scored ≥7 were considered high-quality articles. All non-randomized clinical studies included in the systematic review were of high quality (Table 4).

## 4. Discussion

Treatments based on natural products are regarded as alternative or complementary in certain oral conditions. Indeed, products from the hive such as honey and royal jelly, are used to treat mucositis and other disorders of the oral mucosa [39,40,41].

Although propolis has been traditionally used in folk medicine to treat certain diseases, its mode of action and the chemicals that are responsible for its therapeutic activity remain unknown. It is generally used as a mouthwash at different concentrations (1%, 2.5%, 5%, 10%), although there are other formulations that include oral capsules, gels or cosmetic creams [42].

The aim of this meta-analysis was to determine the effectiveness of propolis in the treatment of periodontal disease. According to the findings, propolis acts better than standardized treatments for certain therapeutic goals such as reducing dental plaque and microbial activity and stabilizing gingival and periodontal indices. It is significant that none of the results included in this review reported harmful or counterproductive effects in participants treated with propolis.

Two of the studies [22,24] addressed the effect of propolis on dental plaque control; four [23,25,26,27] explored the effects of propolis in relation to the reduction of probing pocket depth, inflammation and gingival bleeding; and two [21,28] analyzed the decrease in *Porphyromonas gingivalis* through the use of propolis mouthwashes.

The antimicrobial effect of propolis against periodontal pathogens has been studied in vivo and in vitro [33,43,44]. Nakao and colleagues [21] reported significant improvements in CAL and PPD alongside a trend towards a reduction of *Porphyromonas gingivalis* (a pathogen that plays a key role in periodontal disease) in gingival crevicular fluid (GCF) in patients treated with propolis solutions. Similarly, Yoshimasu and colleagues [45] proved the effectiveness of isolated propolis products such as artepillin C, baccharin and ursolic acid as antimicrobial compounds against *Porphyromonas gingivalis*; artepillin C and bacchatin are bacteriostatics and ursolic acid is a powerful destructor of the bacterial membrane, probably because of its highly lipophilic nature. In a study based on a sample of 20 patients diagnosed with chronic periodontitis, Sanghani and colleagues [28] reported a statistically significant reduction (*p* < 0.05) of *Porphyromonas gingivalis*, *Prevotella intermedia* and *Fusobacterium nucleatum* in periodontal pockets in the group of patients under propolis treatment.

Two of the selected studies analyzed the effect of propolis on dental plaque. Sparabombe and colleagues [22] evaluated its anti-inflammatory effect using a sample of patients with moderate/severe periodontitis who underwent a 3-month treatment with propolis-based mouthwashes, finding a significant improvement in the reduction of plaque buildup and gingival bleeding. Likewise, Piekarz and colleagues [24] reported a significant reduction during the first week of treatment with toothpaste containing ethanolic extract of propolis (*p* < 0.006), and a very significant reduction after 4 weeks of using it (*p* < 0.0002). These results are consistent with those of other studies on the antiplaque and antigingivitic effects of mouthwashes containing other flavonoids and essential oils in individuals with and without periodontal disease [46,47]. Nonetheless, certain studies have shown that certain forms of periodontitis are not associated with plaque and depend exclusively on the individual’s systemic condition [48].

Gingival and periodontal indices and salivary markers of oxidative stress were measured in four of the included studies [23,25,26,27]. Giammarinaro and colleagues [23] studied the efficacy of propolis as compared with chlorhexidine in a sample of 40 patients suffering from gingivitis, finding no significant differences between the control and the experimental group in probing pocket depth (PPD), bleeding on probing (BoP) and plaque index (PI); however, the patients treated with propolis achieved better results in terms of oxidative stress markers in the saliva, with considerable improvement in their periodontal health. Different studies have related oxidative stress in the saliva and the progression of periodontal disease. The main enzymatic antioxidants which have been widely studied in the gingival fluid, saliva and blood serum of patients with periodontitis are superoxide dismutase, glutathione peroxidase and catalase. The activity of these enzymes in gum tissue, gum fluid, saliva and blood serum during the different types of periodontitis (chronic or aggressive) is quite uneven. Despite the fact that Toczewska and colleagues reported that activity in gingival tissue is usually high [49], Tartaglia and colleagues [50], in a preliminary study, found that antioxidant levels in the saliva are reduced in patients with periodontal disease.

Likewise, Miricescu and colleagues [51] found high levels of oxidative stress associated with alveolar bone loss in the saliva of patients with periodontal disease. Likewise, other authors have suggested that patients suffering from this condition are more likely to experience oxidative stress imbalance and have reported that such a situation would be a consequence of periodontitis [52,53,54]. A study on test animals conducted by Aghel and colleagues [55] also proved the beneficial effect of propolis on saliva antioxidants.

The study by Sharkawy and colleagues [26] was the only one where propolis was used as a dietary supplement. It provided a comparison of the use of a placebo and the ingestion of 400 mg of propolis in patients with long-standing diabetes mellitus associated with periodontitis, reporting a significant reduction in periodontal parameters. The group treated with propolis showed a greater reduction in pocket depth (PD) and an increase in CAL as compared with the control group, probably because of the anti-inflammatory, antimicrobial and antioxidant activity of propolis. Interestingly, the use of propolis as complementary to oral hygiene revealed similar results in other studies [56,57].

Pérez de Andrade and colleagues, and Anauate-Netto and colleagues [25,27] reported a reduction in gingival inflammation and probing depth in patients treated with propolis solution mouthwashes as compared with those using 0.12% chlorhexidine or saline mouthwashes.

In general, the anti-inflammatory and antimicrobial properties of propolis have been well documented [56] and it will eventually be possible to explain any type of clinical result, either in healthy or diseased individuals, through the analysis of the oral microbiome [58,59,60].

Nevertheless, this study has a series of limitations, especially because of the heterogeneity found in the measurement of periodontal disease parameters such as plaque or bleeding, loss of clinical attachment, patient hygiene or the oxidative stress of the saliva. We only found unanimity among studies in the measurement in millimeters of probing depth, although two of the studies included did not measure this parameter [24,27]. Bleeding was measured using a variety of indices and assessment criteria: two studies did not evaluate gingival bleeding [24,25], two used the Bleeding on Probing (BoP) index [21,23], another used the Papillary Bleeding Score (PBS) [27], another the Eastman Interdental Bleeding Index (EIBI) [26] and others used unspecified bleeding indices [22,28].

In relation to plaque index, four studies used the Plaque Index (PI) [23,24,25,26], one used the Plaque Control Record (PCR) [21] and another [22] used the Plaque Score (PS); another two studies did not measure plaque [27,28]. An additional factor that is relevant to the assessment of periodontal disease is the Gingival Index (GI), which was only assessed in two studies [25,28]. It should also be noted that certain studies used different indices to measure the parameters and did not specify the criteria used for measurement with each of the indices, which hinders the interpretation of results. Others provided the data concerning the assessment of these indices at the beginning of the study but did not provide numerical data in reassessments, only showing graphs or mentioning that the parameters had been measured but providing no data. There were also discrepancies regarding assessment timing, some studies testing after 1 month and others after 3. Regarding oxidative stress, despite this being considered a predictable and measurable value related to a severe inflammatory state and a marker of the risk of periodontal disease [49,50], only the study by Giammarinaro and colleagues [23] dealt with this situation. All these factors hindered data analysis and precluded their inclusion for quantitative analysis.

For all these reasons, the authors recommend that the results be interpreted with caution, mainly due to the small number of selected studies and the small sample sizes used in each of them.

It would be advisable to perform a larger number of randomized clinical trials comparing the use of propolis with a placebo or chlorhexidine, using unified criteria, to assess periodontal parameters (plaque, bleeding, hygiene, dental mobility…) unified research indices and test timing for such parameters and structured, and standardized guidelines and measures in product administration, which could guarantee reliable and predictable results. Likewise, it would be advisable to reduce the bias of selective disclosure of results and for studies to provide the data related to all the assessed parameters, even if as annexed material to the published study.

## 5. Conclusions

Bearing in mind the limitations mentioned above, it can be concluded that propolis is safe to use and can enhance the results of periodontal disease treatment. Propolis-based therapies are likely to become an alternative treatment option in periodontal diseases and during supportive periodontal therapy. Nonetheless, for these conclusions to be definitely confirmed, further well-designed research, with broader samples, standardized protocols and long-term follow-up to ensure reliable results, is required.

## Figures and Tables

**Table 1:** Jadad quality score of randomized controlled trials included in the meta-analysis.

Study and Year	Randomization	Blinding	Dropouts	Total Score
Nakao et al. 2019 [21]	2	2	1	5
Sparabombe et al. 2019 [22]	2	1	1	4
Giammarinaro et al. 2018 [23]	2	1	1	4
Piekarz et al. 2017 [24]	2	1	1	4
Perez de Andrade et al. 2017 [25]	1	2	1	4
Sharkawy et al. 2016 [26]	2	1	1	4
Anauate-Netto et al. 2014 [27]	2	1	1	4
Sanghani et al. 2014 [28]	2	1	1	4

Each study was assigned a score of 0–5. Mode value: 4.1 ± 0.35.

**Table 2:** Characteristics of randomized clinical studies.

Study and Aim	Participants	Interventions	Outcomes
Nakao et al. 2019.	24 patients with moderate to severe chronic periodontitis.	Propolis ointments were administered to each study group (three times at 1-month intervals) to a tooth with periodontal pockets ≥ 5 mm without local anesthesia. The deepest pocket in the mouth of each subject was chosen.	Treatment with propolis significantly improved PPD and CAL. Reduction of *P. gingivalis* in the gingival crevicular fluid.
Aim: clinical applicability of propolis as an alternative/adjuvant therapy against periodontitis [21].			
Sparabombe et al. 2019.	40 patients with periodontitis.	For 3 months, a polyherbal mouthwash (propolis resin extract, *Plantago lanceolata*, *Salvia*) was prescribed to the test group and a placebo mouthwash to the control group.	Polyherbal mouthwash for 3 months
Aim: evaluate the anti-inflammatory effect of polyherbal mouthwash (propolis resin extract, Plantago lanceolata, Salvia)			reduced inflammation and plaque
in patients with periodontitis [22].			accumulation. Beneficial effect in patients with moderate or severe periodontitis.
Giammarinaro et al. 2018.	40 patients with gingivitis, PPD < 3 mm.	Hydroalcoholic propolis solution (6%) as a mouthwash twice a day for 2 weeks. No propolis in the control group.	Test patients (propolis) had better results in reducing oxidative stress.
Aim: evaluate the effectiveness of a propolis and herbal formula, compared with chlorhexidine-based formulas [23].			
Piekarz et al. 2017	51 patients.	Both groups brushed their teeth twice a day with the received toothpaste for 2 min. Evaluation of the OHI, API and SBI indices, and collection of material for microbiological examination were carried out at the initial visit, at 7 days and at 4 weeks.	Significant reduction in the PI and the SBI in the propolis group. *Candida albicans* was eradicated in the group of patients using the active preparation. Bacteria responsible for the development of gingivitis were eradicated in the study group.
Aim: evaluate toothpaste with active ingredients of plant origin, ethanolic extract of propolis and tea tree oil on the microbiome compared with patients treated with preventive procedures [24].	the study group received toothpaste with ethanolic extract of propolis. The control group received the placebo.		
Perez de Andrade et al. 2017.	18 patients diagnosed with mild to moderate and moderate to severe chronic periodontal disease, with PP ≥ 5 mm deep in uniradicular teeth.	Hydroalcoholic solution of propolis extract 20%.	Probing was reduced with irrigation of 20% propolis extract hydroalcoholic solution as an adjunct in periodontal treatment compared with the control (saline solution).
Aim: evaluate the effect of subgingival irrigation of periodontal pockets with a hydroalcoholic solution of 20% propolis extract as a complement to periodontal therapy [25].			
Sharkawy et al. 2016.	Patients with Type 2 diabetes, with chronic periodontitis with PPD on probing and clinical attachment loss ≥ 5 mm with detectable bleeding on probing in at least one site of each sextant.	Propolis and corresponding placebo capsules. The patients were instructed to take only one capsule per day. All people received SRP.	PD reduction and the increase in CAL were significantly greater in the propolis group than in the placebo group at 3 and 6 months.
Aim: evaluate propolis supplementation in individuals with chronic periodontitis and Type 2 diabetes mellitus who received SRP [26].			
Anauate-Netto et al. 2014.	60 participants;	Groups (1) 2% propolis, (2) 0.12% chlorhexidine and (3) placebo; two rinses a day for 28 days. Papillary bleeding was measured at the beginning of the study and 28 days later.	Reduction in papillary bleeding for the propolis group only.
Aim: compare the effects of propolis and chlorhexidine mouthrinses on gingival health [27].	three groups		
Sanghani et al. 2014.	20 patients; two groups.	Propolis (not exposed to the oral cavity) was placed on the test sites after completing the SRP. The clinical parameters were evaluated at 15 days and 1 month after treatment.	Reduction of GI, BI, PPD and CAL in the test group treated with scaling and root planing and propolis.
Aim: clinical and microbiological evaluation of the subgingival propolis extract as a complement to SRP in the treatment of periodontitis [28].			Lower prevalence of Porphyromona gingivalis, Porphyromona intermedia and Fusobacterium nucleatum as compared with the control group.

PP, periodontal pockets; PPD, probing pocket depth; CAL, clinical attachment levels; OHI, Oral Hygiene Index; API, Approximal Plaque Index; SBI, Sulcus Bleeding Index; PD, pocket depth; GI, Gingival Index; BI, Bleeding Index; PI, Plaque Index; SRP, scaling and root planning.

**Table 3:** Characteristics of Non-Randomized Studies.

Study and Aim	Participants	Interventions	Outcomes	Experimental Propolis Group *p*-Values
Mohsin et al. 2015 Aim: to evaluate the antibacterial efficacy of a propolis-based toothpaste on *streptococcus mutans* that colonized the oral cavity of young patients [32].	30	Subjects were instructed to brush once a day for 3 min for a period of 4 weeks with propolis toothpaste. After 24 h of oral prophylaxis, reference samples were collected.	Statistically significant reduction in mean value of mutant streptococci after 4 weeks compared with the baseline.	1st week *p* = 0.000; 4th week *p* = 0.000.
Coutinho et al. 2012 Aim: to evaluate the effects of subgingival irrigation with propolis extract in deep periodontal pockets by means of clinical and microbiological parameters [33].	20	Subgingival plaque sampling was performed at the beginning of the study and root scaling and planing. Two weeks later, the selected periodontal areas underwent the following treatments: irrigation with a hydroalcoholic solution of propolis extract (Group A), irrigation with a placebo (Group B) or no additional treatment (Group C).	Decrease in Group A anaerobic bacteria compared with the other groups. *Porphyromona gingivalis*: minor levels in test group.	Group A (propolis). A decrease in the total viable counts of anaerobic bacteria; *p* = 0.007.
Tanasiewicz et al. 2012 Aim: influence of the application of toothpaste with 3% ethanol–propolis extract on the state of the oral cavity [34].	80	Pastes/gels: toothpaste with propolis, toothpaste without propolis, gel with propolis and gel without propolis.	Efficacy of preparations containing 3% ethanolic propolis extract in all groups.	API after 8 weeks compared with the first week; *p* = 0.0679 OHI: not statistically significant.
Pereira et al. 2011. Aim: clinical efficacy of a 5.0% Brazilian green propolis mouthwash for plaque and gingivitis control [35].	25	Subjects were instructed to brush their teeth and rinse with 10 mL of 5% green propolis twice daily.	Evidence of the efficacy of alcohol-free mouthwash containing 5% Brazilian green propolis for plaque and gingivitis control.	GI at 45 and 90 days: reduction of gingivitis greater than 40%, statistically significant; *p* < 0.05. PI at 45 and 90 days: reduction in plaque index, statistically significant; *p* < 0.05.
Gebaraa et al. 2003 Aim: to evaluate subgingival irrigation with propolis extract [36].	20	Group A: irrigation with propolis extract twice a week for 2 weeks. Group B: irrigation with 3 mL of a placebo. Group C (control group): no treatment.	Decrease in anaerobic bacteria and increase in *P. gingivalis*. Absence of bleeding on probing at the end of the study.	Decrease in total counts of anaerobic bacteria; *p* = 0.007. Increase in sites with low levels of *Porphyromonas gingivalis*; *p* = 0.005.

API, Approximal Plaque Index; OHI, Oral Hygiene Index; PI, Plaque Index; GI, Gingival Index.

**Table 4:** The Newcastle–Ottawa Scale (NOS).

First Author, Publication Year	Quality Evaluation	Case definition	Representativeness	Selection of Controls	Definition of Controls	Comparability	Ascertainment of Exposure	Same Method?	Non-Response Rate	Score
Mohsin et al. 2015 [32]	1	1	1	1	1	1	0	1	0	7
Coutinho et al. 2012 [33]	1	1	1	1	1	1	1	1	0	8
Tanasiewicz et al. 2012 [34]	1	1	1	1	1	1	0	1	1	8
Pereira et al. 2011 [35]	1	1	1	1	1	1	0	1	0	7
Gebaraa et al. 2003 [36]	1	1	1	1	1	1	1	1	0	8

Each study was assigned a score of 0–9. Mode value: 7.5 ± 0.54.

## Data Availability

Not applicable.

## Supplementary Materials

The following are available online at https://www.mdpi.com/2076-3921/10/2/269/s1, Table S1: PRISMA checklist.

## Abbreviations

ROS	Reactive oxygen substances
OS	Oxidative Stress
SAT	Salivary antioxidant test
IL	Interleukin
TNF-α	Tumor Necrosis Factor α
PP	Periodontal pockets
PPD	Probing pocket depth
PI	Plaque Index
PS	Plaque Score
PCR	Plaque Control Record
OHI	Oral Hygiene Index
API	Approximal Plaque Index
SBI	Sulcus Bleeding Index
PD	Pocket depth
GI	Gingival Index
BI	Bleeding Index
BoP	Bleeding on Probing
CAL	Clinical attachment level
SRP	Scaling and root planing
PBS	Papillary Bleeding Score
NOS	Newcastle–Ottawa Scale
GCF	Gingival crevicular fluid
EIBI	Eastman Interdental Bleeding Index
